# Bovine brucellosis – a comprehensive review

**DOI:** 10.1080/01652176.2020.1868616

**Published:** 2021-01-18

**Authors:** Sandip Kumar Khurana, Anju Sehrawat, Ruchi Tiwari, Minakshi Prasad, Baldev Gulati, Muhammad Zubair Shabbir, Rajesh Chhabra, Kumaragurubaran Karthik, Shailesh Kumar Patel, Mamta Pathak, Mohd. Iqbal Yatoo, Vivek Kumar Gupta, Kuldeep Dhama, Ranjit Sah, Wanpen Chaicumpa

**Affiliations:** a ICAR-Central Institute for Research on Buffaloes, Hisar, India; b Department of Veterinary Microbiology and Immunology, College of Veterinary Sciences, UP Pandit Deen Dayal Upadyaya Pashu Chikitsa Vigyan Vishwavidyalya Evam Go-Anusandhan Sansthan (DUVASU), Mathura, Uttar Pradesh, India; c Department of Animal Biotechnology, College of Veterinary Sciences, Lala Lajpat Rai University of Veterinary and Animal Sciences (LUVAS), Hisar, India; d ICAR-National Research Centre on Equine, Hisar, India; e Quality Operations Laboratory, University of Veterinary and Animal Sciences, Lahore, Pakistan; f Department of Veterinary Microbiology, College of Veterinary Sciences, Lala Lajpat Rai University of Veterinary and Animal Sciences (LUVAS), Hisar, India; g Central University Laboratory, Tamil Nadu Veterinary and Animal Sciences University, Chennai, Tamilnadu, India; h Division of Pathology, ICAR-Indian Veterinary Research Institute, Izatnagar, Bareilly, India; i Division of Veterinary Clinical Complex, Faculty of Veterinary Sciences and Animal Husbandry, Sher-E-Kashmir University of Agricultural Sciences and Technology of Kashmir, Srinagar, Jammu and Kashmir, India; j Centre for Animal Disease Research and Diagnosis, ICAR-Indian Veterinary Research Institute, Izatnagar, Bareilly, India; k Department of Microbiology, Tribhuvan University Teaching Hospital, Institute of Medicine, Kathmandu, Nepal; l Center of Research Excellence on Therapeutic Proteins and Antibody Engineering, Department of Parasitology, Faculty of Medicine Siriraj Hospital, Mahidol University, Bangkok, Thailand

**Keywords:** Brucellosis, bovine, epidemiology, pathobiology, diagnosis, vaccine, treatment, prevention, control

## Abstract

Brucellosis is a zoonotic disease of great animal welfare and economic implications worldwide known since ancient times. The emergence of brucellosis in new areas as well as transmission of brucellosis from wild and domestic animals is of great significance in terms of new epidemiological dimensions. Brucellosis poses a major public health threat by the consumption of non-pasteurized milk and milk products produced by unhygienic dairy farms in endemic areas. Regular and meticulous surveillance is essentially required to determine the true picture of brucellosis especially in areas with continuous high prevalence. Additionally, international migration of humans, animals and trade of animal products has created a challenge for disease spread and diagnosis in non-endemic areas. Isolation and identification remain the gold standard test, which requires expertise. The advancement in diagnostic strategies coupled with screening of newly introduced animals is warranted to control the disease. Of note, the diagnostic value of miRNAs for appropriate detection of *B. abortus* infection has been shown. The most widely used vaccine strains to protect against *Brucella* infection and related abortions in cattle are strain 19 and RB51. Moreover, it is very important to note that no vaccine, which is highly protective, safe and effective is available either for bovines or human beings. Research results encourage the use of bacteriophage lysates in treatment of bovine brucellosis. One Health approach can aid in control of this disease, both in animals and man.

## 1. Introduction

Brucellosis is a bacterial disease associated with evolution of agricultural society, where animal husbandry is an integral part, with worldwide distribution. It is considered as one of the most prevalent zoonosis by Food and Agriculture Organization and World Health Organization (Schelling et al. 2003; WHO 2005, 2012; Corbel 2006). Office International des Epizooties (OIE) declares brucellosis as multiple species disease, infection and infestation (OIE 2018). The etiological agent of bovine brucellosis is a Gram-negative coccobacillus, *Brucella abortus* and occasionally by *Brucella melitensis* and *Brucella suis* (Moreno and Moriyon 2002; OIE 2016; CFSPH 2018a, 2018b). Human brucellosis is popularly known as undulant fever, Crimean fever, Mediterranean fever, remitting fever, Maltese fever, goat fever, Gibraltar fever and bovine brucellosis is called as contagious abortion or Bang’s disease (Hayoun et al. 2020). *Brucella* species are among those pathogenic bacteria which have propensity to adapt to new host and they can either be naturally transmitted to their primary hosts by direct or indirect contact or sometimes inadvertently to other susceptible hosts (Moreno 2014). Mixed farming of cows, buffaloes, sheep and goats has increased the risk of brucellosis where small ruminants act as primary hosts for *B. melitensis* and cattle as spillover host (El-Wahab et al. 2019). In India, brucellosis causes an average loss of US$18.2 per buffalo followed by 6.8 per cattle, 0.7 per sheep, 0.6 per pig and 0.5 per goat (Singh et al. 2015). In this context, lack of enough awareness in public, safe husbandry practices, trading the infected animals and huge economic burden of diagnosis, vaccination and management have led to the persistence of brucellosis in India (Machavarapu et al. 2019).

Effective control strategies of this disease include surveillance, prevention of transmission and controlling the reservoir of infection by different methods including culling (Rahman et al. 2011; ; Durrani et al. 2020). Some countries have controlled *Brucella* infection up to certain extent by implementing the strict immunization protocols such as use of suitable smooth live vaccines, reliable diagnostic tools, mass vaccination of large population, along with consistent culling of *Brucella*-positive animals. If proper vaccination and accurate diagnosis will not be performed, then in the absence of competent immune animals, disease may aggravate due to enhanced virulence, host jumping and wider transmission in different species (Moreno 2014). Vaccination of animals is recommended in highly endemic areas. *B. abortus* strains19 and RB51 are considered as effective attenuated vaccines against infection by *B. abortus* (Dorneles et al. 2015).

In earlier times, when domestic animals were reared in close vicinity of animal owners and handlers, any loophole in the management of animals along with consumption of unsafe dairy or other animal products were major factors for spread of bovine brucellosis and its zoonotic form in humans. Not only domestication of animals, but anthropogenic adaptation of wild animals also provoked this pathogen to widen its host range and jumping from one host to another with possible cross-species transmission. With the passage of time, brucellosis has become a disease causing serious economic losses, which is capable of affecting many species of animals as well as humans owing to the genetic adaptation of the pathogen against a variety of immune defense mechanisms of different hosts. However, humans act as dead-end host and brucellosis occurs with more severe clinical manifestation in man (Moreno 2014). Considering the anthropo-zoonotic potential of brucellosis, approximately 50,000 human cases were annually reported around the globe (Pappas, Papadimitriou et al. 2006). The main portal of transmission to human beings is through raw, improperly pasteurized or unpasteurized dairy products and contact with infected tissues or secretions (Moreno 2014).

This review describes brucellosis with a special focus on bovine brucellosis, its etiology, epidemiology, pathobiology, human health concerns and zoonotic threats, trends and advances in its diagnosis, vaccines, treatment, control and prevention for countering this important disease.

## 2. Historical background

The prevalence of brucellosis was reported in the Mediterranean region and it was historically related with war campaigns. A British army surgeon, George Cleghorn, documented details of disease in the year 1751 in his literature with the title ‘Observations on the Epidemical Diseases in Minorca from the Year 1744 to 1749’ (Hayoun et al. 2020). The disease was described as a separate clinical entity as early as during Crimean war on the island of Malta. The disease was described in detail in the year 1886 by Sir David Bruce, Hughes and Sir Themistocles Zammit (Wyatt 2013). *B. abortus* was firstly discovered by Bernhard Bang, which is known to cause undulant fever in human beings and abortions in cattle (Bang 1897). Traum and Huddleson recovered *B. suis* from swine which is also reported to cause brucellosis in human beings. Evans revealed that *Micrococcus melitensis* (*Brucella melitensis*) isolated from cows and pigs belonged to same genus and nomenclature of genus as *Brucella* was suggested in honor of army Major-General Sir David Bruce (Young 1995). *B. neotomae* was isolated from rat by Stoenner and Lackman (Mantur, Akki et al. 2007; Mantur, Amarnath et al., 2007). *B. canis* was discovered from dogs by Carmicheal and Bruner. *B. pinnipedialis* and *B. ceti* are comparatively newer *Brucellae* isolated from marine mammals during the last decade and could be a potential zoonotic threat in future (Sohn et al. 2003; McDonald et al. 2006). *B. microti* is reported from terrestrial animals (Scholz et al. 2008). The recovery of distinct *Brucella* strains from marine mammals and human beings recently indicates the significance of zoonotic transmission (El-Sayed and Awad 2018).

## 3. The bacterium

Taxonomically, *Brucellae* come under *α*–2 subdivision of *Proteobacteria* (Yanagi and Yamasato 1993). They are Gram-negative, aerobic, facultative intracellular rods or coccobacilli, which lack capsules, endospores or native plasmids. The bacterium has a diameter of 0.5–0.7 µm and has 0.6–1.5 µm length, partial acid fast with oxidase, catalase, nitrate reductase and urease activity. The brucellae are able to survive freezing and thawing, but are susceptible to most of the common disinfectants. The bacterium remains viable in environment for months especially in cool and wet conditions. Pasteurization can effectively kill *Brucella* in milk. Though they are non-motile, yet they have all the genes except the genes required to form a flagellum (Fretin et al. 2005).

A total of six classical and seven novel *Brucella* species have been recognized from a wide spectrum of susceptible hosts. Species affecting terrestrial animals are seven in number including *B. abortus*, *B. melitensis*, *B. suis*, *B. ovis*, *B. canis*, *B. neotomae* and *B. microti* (Scholz et al. 2008); two other species, *B. ceti* and *B. pinnipedialis* affect marine mammals (Foster et al. 2007). *B. papionis* isolated from baboons and *B. vulpis* from red foxes were also added to the list of genus *Brucella* (Scholz et al. 2016). Seven biovars have been recognized for *B. abortus*, three for *B. melitensis* and five for *B. suis*. Rest of the species has not been characterized into biovars. The *Brucella* nomenclature is based on the principal host species (Verger et al. 1987). Reports also document the isolation of 36 atypical *Brucella* spp. from frogs (Scholz et al. 2016; Al Dahouk et al. 2017). As the list of species increases, it is essential to identify better prevention measures to control the spread of disease to man.

### 3.1. Genome

The genomes of all *Brucella* species are having similar size and genome atlas (Sriranganathan et al. 2009), with average genome size of approximately 3.29 Mb consisting of two circular chromosomes. Chromosome I is approximately 2.11 Mb and chromosome II is about 1.18 Mb. The G + C content of chromosome I is 57.2% and chromosome II is 57.3% (Halling et al. 2005). The classic virulence genes for plasmids, capsules, pili or exotoxins are absent in *Brucella* species. A draft genome sequence of *B. abortus* SKN13, isolated from placenta of aborted cattle from Gujarat state of India has proved very useful in providing insight into comparative genomic analysis of *Brucella* strains from India (Chauhan et al. 2016).

*Brucella* isolates in Uganda have been molecularly characterized from cow milk (Mugizi et al. 2015). A genomic monomorphism was found in isolates and showed significant genetic variation when compared with other *B. abortus* biovars from Africa and other countries of the globe. Sankarasubramanian et al. (2017) focused on genome-wide single nucleotide polymorphisms (SNP) based-genome-wide association studies for identification of the genetic determinants in *Brucella* species and could identify 143 species-specific SNPs in *B. abortus* conserved in 311 *B. abortus* genomes, of which as many as 141 SNPs were confined in the positively significant SNPs. In case of *B. melitensis,* out of identified 383 species-specific SNPs in 132 genomes, 379 species-specific SNPs were found having positive association. These species-specific SNPs in genomes could affect host adaptation and also could be responsible for speciation. Sharma, Sunita et al. (2015) reported the draft genome sequences of two *B. abortus* strains from cattle (LMN1) and pig (LMN2), showing novel regions having significant similarity to phages.

### 3.2. Antigenic determinants

The outer cell membrane of brucellae is akin to that of Gram-negative bacteria. There are A and M smooth lipopolysaccharides (LPS) surface antigens; the A antigen is major antigen in *B. abortus* and *B. suis*, whereas the M antigen predominates in *B. melitensis*. These LPS are principal virulence factors as well as target for many immunological tests. Some outer and inner membranes along with cytoplasmic and periplasmic proteins also play a significant role in multiple cellular activities (Meikle et al. 1989). Outer membrane proteins are also useful in development of diagnostic tests.

## 4. Host range

*B. abortus* is the principal *Brucella* organism that infects cattle. However, *B. suis* and *B. melitensis* may also infect cattle (CFSPH 2018a, 2018b). *B. melitensis* and *B. suis* can be transmitted through cow’s milk resulting in human infection (Acha and Szyfres 2003). *B. melitensis* is principally responsible for brucellosis in goats. However, goats may also be infected with *B. abortus* (Lilenbaum et al. 2007). Camels could also be infected by *B. abortus* and *B. melitensis* (Sprague et al. 2012). Camel milk is possibly a major source of human infections in the Middle East countries (Musa et al. 2008). The main causative agent for brucellosis in dogs is *B. canis*; however sporadically, brucellosis in dogs may be caused by *B. abortus*, *B. suis* and *B. melitensis* (Acha and Szyfres 2003).

*B. abortus* has also been reported from Yak and seroprevalence of *Brucella* was studied in Yak (Zeng et al. 2017). A total of 1,523 Yak blood samples were tested using Rose Bengal Plate Test (RBPT) along with a competitive immune-enzymatic assay (c-ELISA) (Zeng et al. 2017). The prevalence of *Brucella* in individual Yak was 2.8% while herd prevalence was reported as 18.2%. The prevalence of brucellosis was found to be much higher in old Yaks in comparison to young Yaks (Zeng et al. 2017). Enström et al. (2017) found 12.4% of sampled animals as seropositive on testing blood samples of 225 cattle in Kenya. However, seroprevalence was found at higher rate in females than male animals (Zeng et al. 2017). The changing geographical distribution of brucellosis as an emerging or re-emerging zoonoses caused a huge economic loss worldwide. A study reported the isolation of *B. melitensis* biovar 3 from cattle, buffaloes, humans and a camel using classical biotyping and Bruce-ladder assay, suggesting its cross-species adaptation to secondary hosts (Sayour et al. 2020). Another study reported the isolation of *B. abortus* from the uterine discharge of apparently healthy female dog and cat housed together in a cattle farm confirms their role as asymptomatic hosts in re-emergence of the bovine brucellosis and its dissemination in farms. Moreover, the study suggested the inclusion of companion animals like dogs and cats in brucellosis surveillance and control program (Wareth et al. 2017).

## 5. Transmission

**Figure 1.**
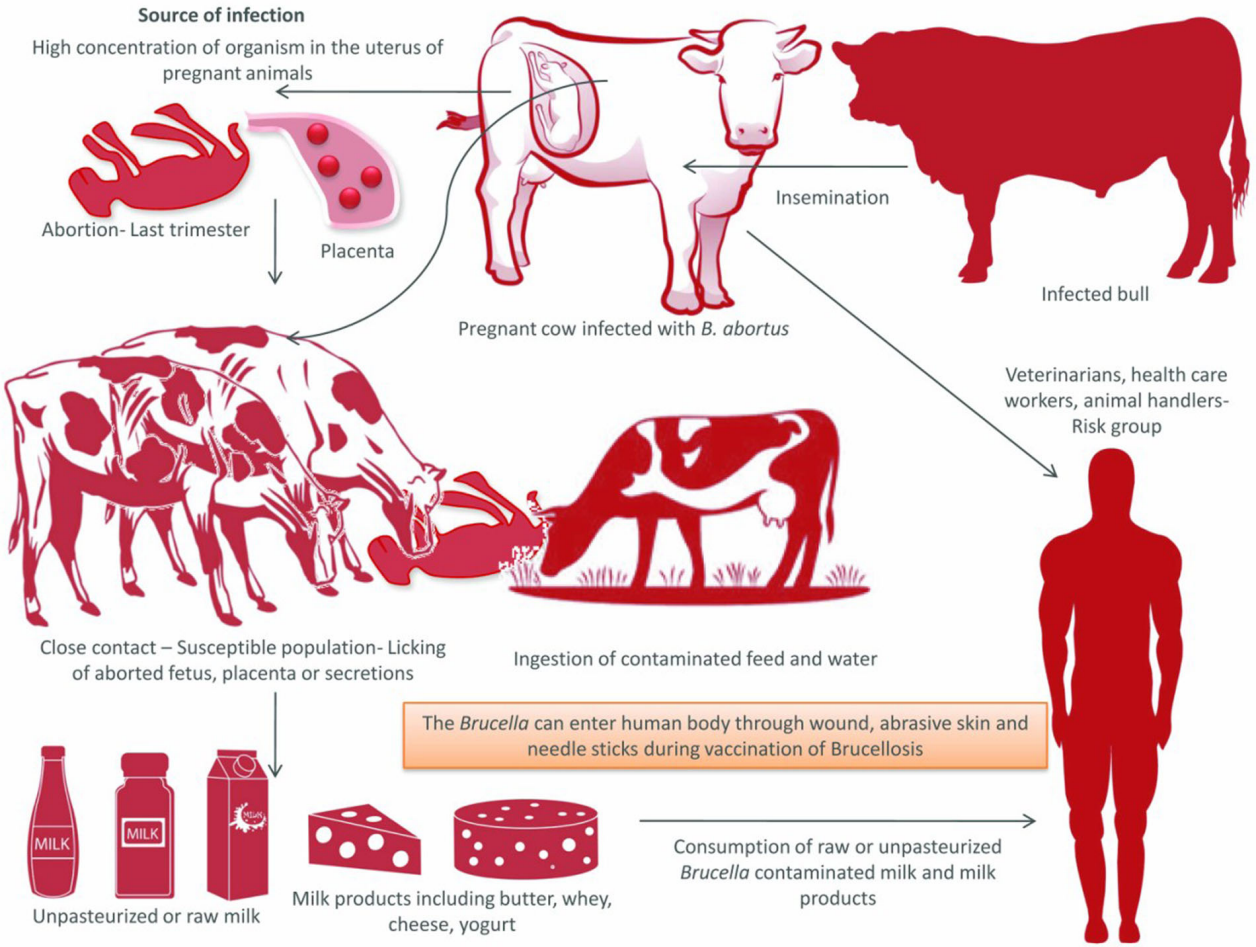
Transmission of Brucellosis. Pregnant cows usually abort in the last trimester of pregnancy. Aborted fetus, placenta, and secretion from uterus act as the source of infection to other animals. Milk and milk products can act as source of infection to man, if consumed unpasteurized. Infected bulls serve as the lifelong source of infection.

*Brucella* can be transmitted *via* horizontal or vertical route (Meltzer et al. 2010). *Brucella* organisms are found in higher concentration in the uterus of pregnant animals. The aborted fetuses, placental membranes and uterine discharges act as main source of infection. Organisms shed in the milk of infected animals may transmit the infection to the newborn. The organism may survive in the environment for months together especially in cold and moist atmosphere. The animals contract the infection by ingestion of contaminated feed and water or by contacting aborted fetuses, fetal membranes and discharges from uterus (Figure 1). Inhalation could also be a mode of transmission. Infected bulls may also spread infection by natural service or artificial insemination from one herd to another (Acha and Szyfers 2001). Tukana and Gummow (2017) described that normal animals sharing common water sources with *Brucella*-positive animals is one of the most important reasons for the spread of brucellosis.

## 6. Geographical distribution

The distribution of brucellosis in different geographies is highly dynamic, with emergence of new areas of infection and re-emergence of infection in areas where infection existed earlier. New areas of prevalence of human brucellosis have emerged in Central Asia and Middle East countries where prevalence is continuously increasing (Pappas, Papadimitriou et al. 2006). This disease is prevalent throughout the world except in Canada, Australia, Cyprus, Norway, Finland, the Netherlands, Denmark, Sweden, New Zealand and United Kingdom. However, Mediterranean Europe, Central and South America, Mexico, Africa, Near East countries, Central Asia, India and Italy are having significant prevalence of brucellosis. Brucellosis is a reportable and notifiable disease in several countries; however, gross under reporting is a glaring problem (CDC 2018). A report considering 19 years (1996–2014) by the World Organization for Animal Health (OIE) regarding 156 countries classified the countries into three groups based on the situation of brucellosis among animals. The three categories are: enzootic for brucellosis: countries that are infected or free of brucellosis for less than 3 years time period, non enzootic for brucellosis: though brucellosis may be present, countries in this category are devoid of disease for a period of 3 years and free of brucellosis: countries devoid of brucellosis throughout the study period of 19 years. The disease free status countries are situated in Europe and Oceania while high prevalence or enzootic countries are present in Central and South America, Africa and parts of Asia (Cárdenas et al. 2019).

Brucellosis is endemic in Western Asia, India, Middle East, Southern Europe and South America (Mantur and Amarnath 2008; Franc et al. 2018). Study in Iran reported that *B. abortus* biovar 3 is the most prevalent biovar (Dadar, Alamian et al. 2019). Reports of low incidence of brucellosis in endemic areas could be due to either inadequate surveillance or under reporting (McDermott and Arimi 2002). Brucellosis is mainly caused by *B. abortus* biovar 1 in water buffaloes in parts of Africa, South America, Brazil, Italy, Pakistan and Egypt (Fosgate, Adesiyun et al. 2002; Megid et al. 2005; Wareth et al. 2014; Ali et al. 2017). In Italy, cattle and water buffalos both are affected by *B. abortus* mainly in southern areas (Garofolo et al. 2017). In Egypt, brucellosis is an endemic problem (Abdelbaset et al. 2018). Reports of *B. melitensis* infection in cattle are pouring which is a major threat in Kuwait, Saudi Arabia, Israel and some southern European countries (Yilma et al. 2016). The epidemiology of this disease remains dynamic and unpredictable as several new strains could emerge and present strains could adapt to new animal species as well as changing situations. Disease is rare in children but in endemic areas, such cases have been reported (Caksen et al. 2002; Mantur, Akki et al. 2004).

Comprehensive reports on the studies from different continents are summarized in the following section.

### 6.1. Africa

Bovine brucellosis was first reported in the African continent in Zimbabwe (1906) followed by Kenya (1914) and South Africa (1915) (Chukwu 1985). However, the epidemiology of the disease in animals as well as humans is not well understood in sub-Saharan countries of African continent. Robust control of brucellosis is not implemented in Africa except in South Africa (McDermott et al. 2013). This disease causes hindrance in import of high yielding dairy breeds and improvement of milk production through cross-breeding (Mustefa and Nicoletti 1993). The prevalence values of brucellosis among indigenous and cross-breed cattle were observed to be 1.1 and 0.6%, and 1.7 and 0% based on RBPT and complement fixation test (CFT), respectively, in Ethiopia (Dirar et al. 2015). The seroprevalence of brucellosis in cattle was conducted in Cameroon, Central Africa, using Rose Bengal Plate Test (RBPT) and indirect-ELISA (Awah-Ndukum et al. 2018). The overall seroprevalence at individual and herd levels were found to be 5.4 and 25.6%, respectively. There was high correlation for managemental factors like region, area, locality, size of the herd and knowledge regarding brucellosis and also factors related with animals like sex and age with seroprevalence of brucellosis (Awah-Ndukum et al. 2018).

Brucellosis is endemic among ruminants and humans in Egypt despite the presence of control programs (Hosein et al. 2018). The annual incidence of human brucellosis is estimated to be 5 to 12.5 million cases in Egypt (Hull and Schumaker 2018). The seroprevalence study of brucellosis in cattle revealed that the overall seroprevalence and seroprevalence at herd level was 2.4 and 45.9%, respectively, in Ethiopia. Moreover, a prevalence of 3.3% was observed in extensive farming system and 1.3% in intensive farming system. Cattle in both systems showed low level of endemicity of brucellosis (Asgedom et al. 2016). Chaka et al. (2018) found herd level prevalence of brucellosis in cattle in Ethiopia at 32% while an overall cattle level prevalence of 9.7% was recorded based on serological tests also. Getachew et al. (2016) conducted surveillance on 278 serum samples for brucellosis in dairy herds in Ethiopia. In this study, sensitivity was reported as 89.6, 96.8 and 94% and specificity was 84.5, 96.3 and 88.5% for RBPT, indirect ELISA, and CFT, respectively. Indirect ELISA was found with the best sensitivity and specificity as compared to both RBPT and CFT (Getachew et al. 2016).

Aworh et al. (2017) screened 376 cattle for *Brucella* infection in Abuja, Nigeria. Out of which 21 were positive with RBPT and 2 with cELISA. The prevalence of brucellosis was low in slaughtered food animals. Seropositivity of brucellosis was highest in Red Sokoto breed of goats in comparison to other breeds. Kamwine et al. (2017) reported a prevalence of 26.5% of *Brucella* in 185 raw milk samples in Uganda using the milk ring test and indirect ELISA. Indirect ELISA-based surveillance of brucellosis was carried out in Tanzania using milk as samples. The study showed a herd prevalence of 44.4% and it also highlighted that the farmers were ready to sell cows that had recent history of abortion. Calf-hood vaccination combined with One Health approach is needed to control the disease in Tanzania (Asakura et al. 2018).

Madut et al. (2018) reported a very high prevalence of brucellosis in cattle and their handlers in Bahr el Ghazal, Sudan, where high level of prevalence in the cattle population was mainly implicated as a source of infection for human subjects and represented a major public health hazard. As per report, the use of real-time PCR and indirect ELISA resulted in detection of *B. melitensis* and *B. abortus* in samples of milk and milk products, respectively.

Tasiame et al. (2016) analyzed blood samples from 178 cattle farmers and 315 cattle for brucellosis in Ghana. Results of RBPT revealed seroprevalence in human and bovine as 10.1 and 22.9%, respectively. However, 86% of bovine cases were confirmed as *Brucella*-positive by cELISA.

### 6.2. South America

A study in Brazil revealed that higher ratio of females in the herd makes the herd more prone to brucellosis. Extensive farming patterns in cattle and procurement of replacement animals from non-certified cattle farms increase the risk of acquiring infection (de Alencar Mota et al. 2016). Recently, bovine samples like uterine discharge, vaginal swab, placenta, milk and aborted fetus were used for isolation of *Brucella* spp. and Multi-locus Variable number tandem-repeat analysis (MLVA) of the isolates were carried out. A total of 10 *B. abortus* biovar 3 was isolated and based on MLVA analysis, 3 isolates were identical to Brazil (Islam et al. 2019).

Carbonero et al. (2018) studied the seroprevalence as well as risk factors for brucellosis in dairy cattle and dairy-beef mixed cattle herds in Ecuador; the true prevalence of *Brucella* seropositivity was 17.0%. The seroprevalence of brucellosis in high-risk areas greatly depend on the husbandry system (Lindahl et al. 2019). The most common risk factors associated with brucellosis includes age of cows, large herd size, source of cattle purchase and geographical presentation of areas. Moreover, *Brucella* seropositivity has been likely associated with abortion. The disease control programs would be beneficial only when these risk factors are addressed strictly (Matope et al. 2011).

Though country level eradication program announced 2 decades back, brucellosis is still prevalent in Colombia. There are various factors that are responsible for the prevalence of brucellosis in this country. To name few are status of *Brucella* spp. other than *B. abortus* is not clear and control measures are targeted towards cattle and not any other animal species. Moreover, a financial investment based-collaborative approach of government, industry and farmers is necessary to encourage effective disease control strategies (Avila-Granados et al. 2019). The mainstay on the path of controlling the disease in livestock includes mere allocation of budget, lack of indemnities to farmers, restricted disease surveillance to *B. abortus* excluding the risk occurrence due to other *Brucella* species and finally more focus on cattle (Avila-Granados et al. 2019).

### 6.3. Asia

Musallam et al. (2015) studied the prevalence, associated risks and distribution patterns of brucellosis in Jordan among ruminant population. The estimated seroprevalence values were reported at 18.1% in cattle herds.

Meta analysis conducted in China to evaluate the prevalence of bovine brucellosis during a ten year period (2008 to 2018) showed that overall prevalence was 1.9%. Northern China had higher prevalence compared to Southern China, and Jilin province had the highest among the provinces with more than 30% (Ran et al. 2019). To study the epidemiological pattern of brucellosis in Hainan province, China, during 2012 to 2017, automatic microbial identification system of Vitek 2 compact was used. Results showed that disease may spread from animals to man and major epidemic strains of *Brucella* were reported to be *B. suis* biovar 3 as well as *B. melitensis* biovar 3 (Wang et al. 2019).

In a study in Pakistan, cattle and buffalo serum samples analyzed using RBPT showed 170 (6.3%) samples and 47 herds (18.6%) to be seropositive for brucellosis (Ali et al. 2017). Seroprevalence was found to be significantly variable depending on different sampling sites. At animal level, replacement of stock, species and sex were significantly correlated to prevalence of brucellosis, whereas insemination method and size of herd were found to be potentially related to prevalence of brucellosis at herd level.

Meta-analysis data showed overall brucellosis prevalence to be 12% or less in India. Lack of effective vaccine strategy and problems associated with culling/slaughter of affected animals are reported to be the reasons for the endemic nature of the disease in India (Deka et al. 2018). Another recent meta-analysis on animal diseases in North Eastern India showed 17% prevalence of bovine brucellosis (Barman et al. 2020). There have been attempts to develop mathematical simulation models to study brucellosis transmission dynamics in an attempt to calibrate the stable levels of bovine brucellosis among cattle population in India (Kang et al. 2014). It was reported in this study that reducing transmission rates will result in lowering the endemically stable prevalence levels of brucellosis in India. High prevalence suggesting a high level of endemicity of brucellosis in India has been reported, with overall national wide average of 5% in cattle, 3% in buffalo, 7.9% in sheep and 2.2% in goat (Renukaradhya et al. 2002). In Punjab, India, the brucellosis infection incidence in buffalo and cattle was reported to be 13.4 and 9.9%, respectively (Dhand et al. 2005). While in 2008, in Punjab, the prevalence of disease in buffalo and cattle increased to 16.4 and 20.7%, respectively, with an overall prevalence of brucellosis at 18.3% (Aulakh et al. 2008). A number of factors related to spread of brucellosis like unhygienic processing of milk and meat, its packaging and handling at different stages determine the zoonotic potential of the disease (Sriranganathan et al. 2009). In India, Pathak et al. (2016) analyzed 481 samples originating from 296 animals including milk, blood, vaginal swabs, vaginal discharges, placental tissue and fetal tissues. Out of these samples, 30.4% samples were found positive for brucellosis by RBPT and 41.6% by indirect ELISA, whereas a seropositivity of 27.0% samples was diagnosed by both tests.

### 6.4. Europe

Though bovine brucellosis was eradicated in Northern part of Ireland in 1980s, it was later reported in 1997 leading to loss of official brucella free status in Northern Ireland. A molecular epidemiological study in Ireland showed that seven clonal complexes were identified based on Multi-locus variable number tandem repeat (VNTR) analysis (MLVA). The study also warranted the use of molecular tools to trace the source of infection (Allen et al. 2015).

A specific resolution on adopting the One Health approach at the World Health Assembly in 2013 has successfully controlled the 17 neglected tropical diseases (NTD). Similarly, adoption of One Health approach in controlling the endemic NTD, like brucellosis is necessary that this needs support from the global community (Mableson et al. 2014).

## 7. Pathogenesis

Various virulence factors, mechanism of evasion from host defense systems and mode of intracellular survival of *Brucella* have been extensively reviewed by Gopalakrishnan et al. (2016). The major pathogenic attributes in brucellosis are factors like LPS, urease, adenine monophosphate, guanine monophosphate, vir B and 24-kDa protein. Genome of *Brucella* is devoid of classical virulence genes which encode for plasmids, pili, exotoxins and capsules (Seleem et al. 2008). The mode of transmission is either through ingestion, inhalation, *via* conjunctiva and through abrasions/wounds in skin. Once entered in the host body, *B. abortus* multiplies in intracellular milieu of phagocytic cells such as macrophages and dendritic cells and when female conceives, the bacteria reach trophoblasts and the mammary gland through circulation, and very expansively multiplies to induce abortion. While in non-pregnant animals, bacteria continue to multiply and shed in environment through various body secretions and excretions (Moreno 2014; de Figueiredo et al. 2015). The *Brucella* are frequently isolated from milk, supra-mammary lymph and iliac lymph nodes, spleen and uterus. However, bones, joints, brain and eyes might also become infected. The bacteria are isolated commonly from genital organs and associated lymph nodes in bulls. A large number of bacteria are excreted in semen during early acute phase, but the excretion decreases gradually later in chronic phase. The bacterial excretion may regularly continue for many years or could be intermittent (Acha and Szyfers 2001). *Brucella* reaches the placenta in females *via* hematogenous route and afterwards to the fetus. The allantoic fluid factors in females stimulate the growth of *Brucella,* thereby making the uterus and reproductive tract of the pregnant female the site of the bacterial predilection. The elevated level of erythritol in the placenta and fetal fluid from fifth month of gestation is thought to be an important factor for abortion in animals (Anderson and Smith 1965).

Erythrophagocytic trophoblasts localize in the placentome in vicinity of chorio-allantoic membrane resulting in rupture of cells and ulcer formation in the chorio-allantoic membrane. Abortion occurs due to the damage inflicated by the bacteria on the placenta and also due to stress induced hormonal changes (Radostits et al. 2000). Perin et al. (2017) analyzed the changes in adenosine deaminase activity and the oxidative stress in brucellosis serologically positive cows in Brazil. It was revealed that there was reduction in the activity of adenosine deaminase as well as catalase in serologically positive animals; simultaneously, an increase in the level of oxidative stress markers along with superoxide dismutase as well as thiobarbituric acid reactive substances was observed in *B. abortus* infected cows. A reduction of adenosine deaminase along with oxidative stress could possibly be related to inflammatory response modulation. As *Brucella* spp., is intracellular pathogens which can survive within the phagocytic cells by using various escape strategies to undermine the host immune defense mechanism, it can progress from acute to chronic and to carrier form in host. Studies have confirmed the role of gene polymorphisms (Amjadi et al. 2019).

## 8. The disease

### 8.1. Clinical signs

Various clinical signs have been described in infected animals, the main manifestation in *B. abortus* infection being reproduction failure in the form of abortion and birth of weak offsprings which remain as carrier in herd. The clinical signs, manifestations and multiple complications in brucellosis in different animal species are firstly related to the reproductive tract. The incubation period could vary from two weeks to months together. Calves could be infected at early stage but no symptoms are seen till they mature. It is manifested by late abortions in pregnant animals, birth of weak calves, lowered fertility, retention of fetal membranes, endometritis and reduction in milk production (Kiros et al. 2016; Abdisa 2018). Abortion rate may vary from 30 to 80% in susceptible herds (Kiros et al. 2016). Calves borne at full-term may die very soon after birth. Fibrinous pleuritis coupled with interstitial pneumonia also appears in newborn calves and also in aborted fetuses (Carvalho Neta et al. 2010).

Male animals show clinical manifestations in the form of orchitis and epididymitis, whereas, hygroma is witnessed in chronic infections (Corbel 1997). Cervical bursitis in cattle has also been reported due to brucellosis (de Macedo et al. 2019). In the seminal vesicles, the acute inflammatory phase is followed by a chronic stage with considerable fibrinoid induration. Areas of dry necrosis develop and become encapsulated by fibrinous tissue, which eventually contracts, often leaving the testicles smaller than normal. In some cases, it may soften with the production of a soft fluctuating lesion containing thin purulent exsudate.

### 8.2. Pathology (Gross and histopathology)

Granulomatous inflammatory lesions are commonly seen in *Brucella* infections in animals often within lymphoid tissues and organs with a major involvement of the mononuclear phagocytic system (MPS). The persistence and localization of bacteria in these tissues follow widespread distribution of the *Brucella* in generalized phase of infection. Abortion in females and infertility in males result from localization of Brucellae within the female and male reproductive tracts (Enright 1990).

Invasion of gravid bovine uterus is marked by characteristic necrotic placentitis, which may be acute and widespread and lead to early death of fetus followed by abortion, or sub-acute or chronic placentitis leading to a late abortion or birth of a live or infected calf. The cotyledons are congested or swollen and covered with yellowish or sticky brownish exudates, which extends deep into the crypts. The inter-cotyledonary areas are thickened, opaque, often look almost leathery and there is often a loss of normal reddish coloured appearance. There is enlargement of liver and spleen in aborted fetus, along with a significant increase in fluids in body cavity. The fetus varies from typical hairless 4–7 months up to one that is fully developed and shows no characteristic lesions (Stableforth and Galloway 1959).

Pneumonitis of a broncho-pneumonia type is noticed in the lungs. In some cases, congestion and fibrinous exudation occur; in other, cellular infiltration of bronchioles, peribronchial tissues, alveoli and perialveolar tissue predominates. Cobblestone lesions on lungs are indicative of brucellosis (Stableforth and Galloway 1959).

## 9. Disease spectrum in humans

Human brucellosis is primarily caused by *B. melitensis* globally*. B. abortus, B. suis* and *B. canis* also cause human brucellosis worldwide (Al-Nassir 2018). Sheep, goats and their products are major sources of *B. melitensis* infection in human beings (Corbel 2006).

Main source of transmission of *B. abortus* to human is through consumption of unpasteurized or raw milk or milk products including butter, whey, cheese, yogurt, ice-cream, *etc*. (Dhanashekar et al. 2012). A study in Turkey revealed that 16.6% of the cow, 6.1% of the goat and 6.1% of the sheep milk and 16.3% of the cheese samples were positive for anti-*Brucella* antibodies using indirect ELISA, while *Brucella* DNA was detected in 18.8, 7.6, 6.1 and 22.5% of cow, goat and sheep milk and cheese samples, respectively (Altun et al. 2016). Infection through raw vegetables, water with fecal contamination and consumption of under cooked animal meat are also reported (Radostits et al. 2000). Sour milk, yogurt and hard cheese result in propionic and lactic fermentation, therefore survival of organism is comparatively less (Corbel 2006). Recently a meta-analysis on brucellosis contamination in dairy products showed that highest prevalence was reported in Southeast Asian region while lowest prevalence in Western Pacific region. The study also showed that increased awareness and increase in countries GDP can reduce the level of *Brucella* sp. contamination in dairy products, thereby preventing the transmission; thus is related to level of poverty also (Dadar et al. 2020).

Brucellosis in man is also considered as an occupational disease of dairy farmers, milking workers, animal handlers, dairy industry workers, slaughter house staff, butchers, hunters, shepherds, laboratory personnel, veterinary assistants, scientists and veterinarians (Walker 1999). Infection through skin wounds may occur in persons working in meat industry, veterinarians and livestock handlers. Inhalation is an important cause of infection in slaughter house personnel (Robson et al. 1993). Laboratory acquired infection is reported as a potential health emergency for the laboratory personnel. Brucellosis is considered a common laboratory-transmitted infection (Bouza et al. 2005; CDC 2008). Brucellosis may be transmitted from wild animals to domestic animals and ultimately to human beings (Cutler et al. 2005). Moreover, a little knowledge on the disease and its transmission among livestock handlers negatively reflect their attitude and practice towards the disease control strategies (El-Wahab et al. 2019).

The pathogen has been classified as a category (B) pathogen possessing the potential to be used as bio-weapon (Seleem et al. 2010). In fact, *B. suis* was the first agent used by American army as a biological agent for biological warfare (Riedel 2004). Laboratory-acquired brucellosis occurs mainly through aerosol (Ergönül 2004).

A woman was diagnosed with brucellosis whose husband suffered with relapse of bacteremia with *B. melitensis* biotype 3 (Vigeant et al. 1995). Human brucellosis may be transmitted between humans, sexually, placental barrier, lactation and tissues such as bone marrow and blood transfusion (Naparstek et al. 1982; Lubani et al. 1988; Mantur et al. 1996; Tikare et al. 2008; Meltzer et al. 2010; Tuon, Gondolfo et al. 2017). Man-to-man transmission of brucellosis is not common; however, outbreaks may be possible indicative of a common source of infection (Chomel et al. 1994; Fosgate, Carpenter et al. 2002).

The most common symptoms of human brucellosis include undulant fever, lack of appetite, weight loss, night sweats, uneasiness, fatigue, chills, insomnia, joint pain, constipation, headaches, myalgia, sexual impotence, nervousness, depression and loss of weight (Koshi et al. 1971; Mousa et al. 1987; Acha and Szyfres 2003; Kochar et al. 2007; Mantur, Amarnath et al. 2007). As the symptoms of brucellosis are often similar to several other common human diseases, this results in under reporting and under diagnosis of disease in humans (Corbel 1997; Maichomo et al. 2000). In human, the disease symptoms depend on the affected site of infection such as encephalitis, meningitis, spondylitis, arthritis, osteitis, orchitis, endocarditis, epidydimitis and prostatitis (Megid et al. 2010; Kiros et al. 2016; Rahdar et al. 2019). Sudden abortions during first or second trimesters are observed in pregnant women (Kurdoglu et al. 2015; Vilchez et al. 2015; Yang et al. 2018). Man-to-man transmission of brucellosis is very uncommon. However, sexual and intrauterine transmission to the infant could also be possible (Khan et al. 2001; Kato et al. 2007). Moreover, infants may get infection from breast-feeding mothers infected with brucellosis (Al-Eissa 1990; Palanduz et al. 2000; Tikare et al. 2008).

Human brucellosis occurs along with recurring complications including arthritis, osteomyelitis, bursitis, discitis and tenosynovitis in 10-85% of patients. In rare situations, spinal brucellosis gets aggravated into epidural abscess, which further deteriorate to permanent neurological defects leading to mortality if not treated on time (Esmaeilnejad-Ganji and Esmaeilnejad-Ganji 2019).

Involvement of lungs is not very rare (Pappas et al. 2003; Mantur et al. 2006). Tsolia et al. (2002) has reported unusual complications like thrombocytopenic purpura and acute facial nerve palsy in two children in Greece. There is a report of acute panniculitis as an unusual manifestation of brucellosis (Tanyel et al. 2008).

*Brucella* infection without any symptoms has been reported in humans. One hundred farm workers in Khartoum North and Omdurman, Sudan, were tested for presence of *Brucella* antibodies and 10% of them were found positive, though they did not have any apparent form of disease (Osman et al. 2015).

Even consumers are prone to have brucellosis from milk samples from brucellosis vaccinated (RB51) cattle, if not pasteurized properly (Ashford et al. 2004). As *B. abortus* RB51 is resistant to rifampin which is a drug of choice against human brucellosis; hence, correct diagnosis and combined therapy need to be formulated (Cossaboom et al. 2018).

One study in Africa revealed that patients with travel history in brucellosis endemic countries were diagnosed with brucellosis after intake of raw camel milk (Rhodes et al. 2016). In another report, a veterinarian exposed to RB51 during vaccination in 2017 at Oregon, USA suffered with symptoms of fever, cough, malaise, myalgia, arthralgia and right upper lobe pneumonia after four days of exposure by needle pierce route (Weese and Jack 2008). In case of human RB51 infections, doxycycline and trimethoprim-sulfamethoxazole (TMP/SMX) combination for 60 days by oral route are recommended (Hatcher et al. 2018). Human infection caused by *B. canis* has been reported (Dentinger et al. 2015). Many times, disease remains undiagnosed in human because it begins with mild fever and no specific symptoms, hence isolation of organism, serological confirmation and molecular tests are of paramount importance (Al Dahouk and Nockler 2011).

## 10. Public health and economic importance

Brucellosis, particularly *B. melitensis*is is thought to be one of the most prevalent re-emerging zoonotic diseases globally with an estimated incidence of more than 50,000 human cases per year (Gwida et al. 2010). The zoonotic importance of brucellosis as zoonosis is increasing owing to tremendous increase in global trade in animal products, rapid deforestation, unplanned and unsustainable development, urbanization, intensive farming, having migratory/nomadic animal husbandry and increased international tours and travel (Memish and Balkhy 2004; Bayeleyegn 2007). Even the exhaustive and advanced surveillance and control measures have not been able to reduce the prevalence of brucellosis in most of the developing countries due to poor hygiene, lack of sanitation, poverty, lack of proper administration and political will (Pappas, Christou et al. 2006).

Brucellosis badly affects livestock welfare and economy. The collective economic losses are the cumulative effect of reduction in the production of milk, abortions, losses of newborn calves resulting from abortions and stillbirths, culling of brucellosis affected animals, hindrance in export and trade of animals, loss of human effort in terms of man-days wasted, veterinary and medical expenses, administrative and governmental expenses on research and control programs (Georgios et al. 2005). Brucellosis patients as well as their family members should be screened regularly in endemic areas (Almuneef et al. 2004; Mantur et al. 2006). Incidence of human brucellosis varies from <0.01 to >200 per 100,000 population in endemic areas globally (Bano and Lone 2015). Six countries comprising of Syria, Saudi Arabia, Oman, Jordan, Iran and Egypt have accounted for more than ninety human brucellosis case reports annually in 1990 (Awad 1998). Brucellosis results in colossal economic losses worldwide both in terms of animal health and production as well as from public health aspects in terms of cost of treatment along with loss of productivity. Bovine brucellosis results in economic losses in countries of Latin America to the tune of approximately US $600 million (Angara et al. 2016). The cost of national brucellosis control and eradication program in USA was of the tune of US $3.5 billion during the year 1934 to 1997 and the cost due to reduction in milk yield and abortions in 1952 alone was estimated to be US $400 million (Sriranganathan et al. 2009).

Kothalawala et al. (2017) investigated the link of socio-economic factors and *Brucella* prevalence in Sri Lanka. Socio-economic parameters like income of family, education level of family members, ethnicity affiliation, experience in farming, and advanced training in animal husbandry techniques were thought to be basic factors as potential farmer level risk factors. Herd size, feeding method, grazing pattern, breeding protocols and methods, and occurrence of abortions at farm were considered as herd factors. The overall seroprevalence of brucellosis was 2.7% at animal level and 9.6% at the herd level (Kothalawala et al. 2017). Poverty level was also highly associated with the occurrence of disease. Grazing practices involving free movement of animals and introduction of animals from outside especially of unknown health status were positively related to brucellosis. A study in Dushanbe, Tajikistan, showed that out of 564 milk samples, 58 samples were positive for brucellosis by real-time PCR. Consumption of unpasteurized milk is a practice in this area and hence the result was of significance as the contaminated milk can transmit brucellosis to man (Lindahl-Rajala et al. 2017). Analysis of blood samples for brucellosis by RBPT, Standard Tube Agglutination Test (STAT) and ELISA from 279 veterinarians in India showed that 53.8% of the samples were positive by IgG-ELISA. Years of service as veterinarian were found to be a risk factor for brucellosis. One interesting finding is the use of personal protective equipment (PPE) was also associated with occurrence of brucellosis, which may be due to use of PPE after infection or improper use of PPE (Proch et al. 2018). Very recent report shows that incidence of brucellosis was higher in man who consumed relatively more of goat milk (Mangtani et al. 2020). Brucellosis was reported in human patients with acute febrile illness in Pakistan. *B. abortus* was found positive in 26 out of 446 blood samples by PCR. Risk factors include contact with affected animals, consumption of unpasteurized milk; females had higher risk compared to males as per the study (Saddique et al. 2019). A study in Kars, Turkey, showed 1.9% prevalence of brucellosis in milk and milk products. Total of 315 samples of raw milk, cheeses and butter were examined for brucellosis by PCR and bacteriological examination. Pasteurization of milk before consumption is essential to prevent transmission of the pathogen to humans (Gulbaz and Kamber 2016). This poses a major public health threat by consuming non-pasteurized milk products produced by unhygienic dairy farms where brucellosis is endemic (Wareth et al. 2014).

## 11. Diagnosis

Epidemiological patterns and related information as well as history of the disease are very important for clinical diagnosis of brucellosis. World Health Organization (WHO) has reported in its factsheet that around millions of cases of brucellosis are accounted every year but actual rate of incidence is still 10–25 times more than the stated number of cases. One important reason behind this condition is lack of distinct guidelines for diagnosis of brucellosis cases. Based upon study of the clinical and diverse serological pattern of disease, researchers have proposed that after acute form of brucellosis immune response is mainly comprised of IgM, secondary immune response is in the form of IgG, which generally gets weaker after improvement of condition and no permanent positivity to IgG antibody is present for longer duration. They showed that such variable serological pattern of disease suggests seven possible clinical subtypes of the disease modulating the epidemiological scenery of brucellosis (Avijgan et al. 2019).

Timely and authentic diagnosis is central to the therapeutic management and control of infection. Detection is mainly done by bacterial culture techniques and by various serological methods which also help in herd screening, surveillance programs and in planning, control and eradication strategies in different geographical locations globally (Dos Santos et al. 2017; Ducrotoy et al. 2018). Bacteriological analysis following identification of suspicious colonies still remains the gold standard diagnostic technique. Although PCR-based methods have been reported to be effective in diagnosing brucellosis in livestock, its failure to distinguish the field strain from the vaccine strain becomes the major drawback. As per study, a novel real-time PCR-based method targeting the outer membrane protein of *B. abortus* reported to differentiate between the virulent and S19 vaccine strain of *B. abortus*, thereby preventing the occurrence of false positive results during monitoring the disease in endemic areas (Kaynak-Onurdag et al. 2016). As per report, the in-housed fluorescence polarization assay (FPA) and competitive ELISA (cELISA) act as a potential diagnostic tool in detecting *B. abortus* S19 post-vaccinal antibodies as compared to Rose Bengal Plate Test (RBPT), indirect ELISA and available commercial cELISA kits (Kalleshamurthy et al. 2020).

Laboratory confirmation from serum samples is essential for confirmatory diagnosis of brucellosis (Young 1995). Several laboratory methods including isolation and identification of the organism, *Brucella* specific antigen and antibody detection methods, and molecular detection are useful in brucellosis diagnosis (Solera et al. 1997; Habtamu et al. 2013; Karthik, Rathore, Thomas, Elamurugan et al. 2014; Karthik, Rathore, Thomas, Arun, Viswas, Agarwal et al. 2014). The basic techniques for brucellosis diagnosis are serologic tests such as detection of antibodies occurring in response to infection by CFT, 2-mercaptoethanol agglutination, Coombs test and Burnet's intradermal test which can identify the state of hypersensitivity of infected subject to *B. abortus* (Galińska and Zagórski 2013). Sometimes, when the load of infection is low or in initial stages of infection, few serological tests fail to detect the infection. Such scenario indicates the need of molecular targets and novel biomarkers for the early and accurate diagnosis of infection to implement proper prophylactic or therapeutic measures. Brucellosis prophylaxis program depends on accurate and precise diagnosis of the disease. However, the RBPT and the CFT could not prevent the false positive results caused by other bacteria sharing smooth lipopolysaccharide (S-LPS) components with *Brucella* spp. Moreover, to avoid this single reaction phenomenon, a *Brucella melitensis* B115-based ELISA resulted in a potential diagnostic test in preventing the unnecessary slaughter of false positive animals (Trotta et al. 2020). MicroRNAs (miRNAs) could also be promising markers to diagnose brucellosis.

### 11.1. Isolation and identification

Isolation of bacterial pathogens is always a confirmatory diagnosis and gold standard. However, disadvantages are the long time required for definitive identification, usually two weeks (Radostits et al. 2000). For isolation of the organism, the most reliable samples in animals are spleen as well as lymph nodes (iliac, mammary as well as prefemoral) during the post mortem. In clinical sample, the viability of organisms is highly essential for the isolation of the organism. From infected animals, the best source of isolation are the uterine discharges as well as aborted fetuses. From aborted fetuses, the samples of choice are contents of stomach, spleen, liver, lungs as well as lymph nodes (Yagupsky 1999).

Direct isolation and culture requires solid media, thereby limiting establishment of non-smooth mutants and development of contaminants in excess. Liquid media are, however, recommended for voluminous samples or for purpose of enrichment. *Brucella* medium base, tryptose (or trypticase)–soy agar (TSA) are the dehydrated basal medium available commercially. Bovine or equine serum (2–5%) must be added for the growth of strains such as *B. abortus* biovar 2. Blood agar base or Columbia agar provides excellent results. Serum–dextrose agar (SDA) or glycerol dextrose agar are other satisfactory media and help in observation of colonial morphology (Alton et al. 1988). Castañeda’s medium (non-selective biphasic medium) is recommended for the isolation of *Brucella* from blood and other body fluids or milk, thereby providing enrichment and prevents interference in biotyping when the organism is grown in broth (Alton et al. 1988; Mantur et al. 2008a). *B. abortus* requires serum and carbon dioxide for growth, whereas it is not required for *B. melitensis*. Selective media like Farrell’s selective medium is however required for avoiding growth of contaminants and such media are used for isolating the bacteria from milk samples. There has also been a report regarding the use of nalidixic acid as well as bacitracin with inhibitory effects on certain strains of *B. melitensis*. Thayer-Martin’s medium can also be used as an alternative (Quinn et al. 1994). Colonial morphology, staining and biochemical characters like catalase, oxidase and urease can aid in identification and confirmation of *Brucella* spp.

Blood culture is confirmatory evidence for brucellosis in human; however, it may give negative result in some brucellosis positive patients (Colmenero et al. 1997). Sensitivity of culture technique is poor in chronic patients. Blood clot culture and lysis centrifugation are promising methods for diagnosis of brucellosis in human as they are faster and sensitive (Mantur and Mangalgi 2004; Mantur, Bidari et al. 2007). Several automated blood culture systems are available which have made the human brucellosis diagnosis even faster (Bannatyne et al. 1997). Bone marrow cultures yield promising results and have been reported as the gold standard by some workers for diagnosis of human brucellosis (Gotuzzo et al. 1986; Mantur et al. 2008a). However, their reproducibility is questionable (Shehabi et al. 1990). The bacteremia could be resulted by several other attributes of mononuclear-phagocytic system also (Mantur et al. 2008a, 2008b).

*Brucella abortus* biovar 3 from dairy cattle was isolated from milk, organs of aborted fetus, fetal membranes and placenta in Tanzania. The primary isolation of *Brucella* species was done on selective serum dextrose agar medium along with Farrell’s medium stained with Gram-staining and were identified by phase contrast microscopy (Mathew et al. 2015). Similarly, in Ethiopia, *Brucella* species were isolated from seropositive cattle with a history of abortion. *B. abortus* was isolated from vaginal swab (8.69%) and placental cotyledon (11.1%). However, no isolate was detected from aborted fetal abomasal contents and milk of animal (Geresu et al. 2016). Modified Agrifood Research and Technology Center of Aragon (CITA) medium (mCITA) was better for selective isolation of *Brucella* spp. compared to Farrell's medium (FM) and modified Thayer Martin (mTM). Nevertheless, Farrell's medium allows inhibition of fungi during isolation; hence mCITA or FM can be used for isolation of *Brucella* spp. (Ledwaba et al. 2020).

Comparative analysis of cultural and serological techniques was done for the diagnosis of brucellosis in 248 cattle of four dairy herds (O’Grady et al. 2014). For bacterial culture, paired supra-mammary, retropharyngeal and internal iliac lymph nodes were subjected to bacteriological analysis while five serological tests employed were microserum agglutination test, indirect ELISA, cELISA, CFT and fluorescence polarization assay. *B. abortus* could be isolated from 86.8% cases. In contrast to this, comparatively lesser (80.9%) animals were detected positive in at least any one serological test while merely 45.2% showed positivity in all five serological tests, although microserum agglutination test and fluorescence polarization assay were found to be comparatively more sensitive out of five serological tests. Overall analysis advocated that along with serological tests, bacterial culture methods should always be encouraged and practiced for confirmation of brucellosis (O’Grady et al. 2014).

### 11.2. Polymerase chain reaction (PCR) assay

PCR is a rapid diagnostic method, which may be applied even on samples of poor quality. This could be used for epidemiological interpretations and analysis as well as for molecular characterization. A number of sequences have been recognized as targets for genus-specific PCR assays for confirmation of *Brucella* species, *viz*., *omp2* and *bcsp31*,16S rRNA and the 16S-23S region (Navarro et al. 2002; Habtamu et al. 2013). A real-time PCR for the authentic diagnosis of *B. abortus*, *B. melitensis* and *B. suis* biovar 1 has been developed (Redkar et al. 2001). The genus *B. abortus* and *B. melitensis* specific primers could detect specific *Brucella* species authentically (Navarro et al. 2004; Neha et al. 2017). A real-time PCR was optimized for detection of various regions of the *Brucella* genome including 16S rRNA, 31-kDa OMP and *IS*711 genetic element. The efficiency of the *IS*711-based PCR for detection of *Brucella* from milk, blood and lymph tissue at the level of 10 gene copies was examined. Blood samples of naturally infected cows were found negative against *B. abortus*; however, milk and lymph tissues were found positive (O’Leary et al. 2006). A more sensitive and specific unique repeat sequence PCR (URS-PCR) has also been validated for confirmatory diagnosis of *B. abortus* and *B. melitensis* (Alamian et al. 2017). PCR is also proven useful in diagnosing relapsing brucellosis, assessing treatment efficacy, identification and differentiation of biovars and biotypes, respectively (Christopher et al. 2010).

Fast and accurate diagnosis of bovine abortion cases caused by *B. abortus* necessitates the use of sensitive, specific and reliable diagnostics. Out of the 103 samples, 28 samples produced 193 bp amplicon specific for *Brucella* genus (Mahajan et al. 2017). The species-specific primers amplified a 498 bp amplicon corresponding to *B. abortus*. PCR and Immunohistochemistry (IHC) were found reliable for the confirmation of bovine brucellosis in aborted fetal tissue and placental cotyledons, whereas serology was useful for detection of *Brucella-*positive animals in a herd. Researchers also designed more accurate two-step PCR for early detection of brucellosis from 39 brucellosis cases and 25 control (healthy) cases. Multiple sequence alignments (MSA) analysis showed that N terminal region of the Omp2 protein was related with highly conserved region of the genome of *Brucella* (Safari et al. 2019).

### 11.3. Serological tests

Serological tests are important for monitoring, surveillance, control and eradication programs worldwide. Antibodies start to appear in the blood in about a week after infection of *Brucella*. The IgM appears first followed by the appearance of IgG. Several serological tests, *viz*., RBPT, standard tube agglutination test (SAT), immune capture agglutination, CFT, milk ring, Coombs test, ELISA and lateral flow assay (LFA) are frequently employed to diagnose brucellosis (Lucero et al. 2003). Assays like RBPT and LFA can be performed at the point of sample collection; thereby reducing the time required for diagnosis. A surveillance study in rural settings of Western Uganda reported that RBPT and LFA can be used for accurate diagnosis of brucellosis (Ezama et al. 2018).

### 11.4. Rose Bengal Plate Test (RBPT)

Rose Bengal Plate Test (RBPT) is helpful in quick confirmation of neuro-brucellosis, arthritis, epididymitis, orchitis and hydrocele (Mantur et al. 2006). The sensitivity of RBPT is very high, but it is less specific (Barroso et al. 2002). On testing 384 serum samples from cattle in Southern Ethiopia for the detection of *Brucella* specific antibodies using RBPT, overall seroprevalence of 4% was reported. Abortion and retained fetal membrane (RFM) were found significantly associated with seropositivity (Yilma 2016). Similarly, a report of RBPT revealed an overall seroprevalence of *Brucella* in the small dairy unit and conventional setting cattle management system as 4.1 and 7.3%, respectively, in Tanzania (Swai and Schoonman 2010). Indeed, RBPT has a better relative sensitivity and specificity in comparison to the SAT and CFT for human samples (Teng et al. 2017).

### 11.5. Complement fixation test (CFT)

Complement fixation test (CFT) is a very specific test that can detect IgM and IgG1 antibodies. However, antibodies of the IgG2 type impede complement fixation resulting in exhibition of false negative results. The CFT accounts for quantitative measurement of more of the IgG1 type antibodies than the IgM type antibodies, as the inactivation process results in partial destruction of IgM antibodies. CFT is considered better for control and surveillance programs for brucellosis (Buchanan and Faber 1980). CFT was performed on sera from cattle and buffaloes vaccinated with RB51 vaccine, *B. canis* infected dogs and *B. ovis* infected sheep using hot saline extract (HSE), RB51 and B115 as antigens. The B115 CFT was found to be very sensitive and specific in detecting rough strain antibodies as compared to RB51 and HSE-CFT. As such, *B. melitensis* B115 is promising antigen for CFT for detecting antibodies against rough strains of *Brucella* (Adone et al. 2008).

### 11.6. Standard tube agglutination test (SAT)

SAT is the most popular diagnostic tool used worldwide for the diagnosis of brucellosis due to its simplicity and economy. SAT accounts for aggregated quantity of IgM and IgG, while the quantity of specific IgG is measured by 2-mercaptoethanol (2ME) treatment of serum sample. IgG antibodies are important for detection of active brucellosis and is an excellent indicator of active brucellosis. A rapid decline in the titer of IgG antibodies is indicator of successful treatment. Persistence of SAT antibodies in some successfully treated patients indicate over diagnosis of human brucellosis resulting in wrong treatment (Almuneef and Memish 2002; Mantur et al. 2006).

The limitations of this test include that it is not able to diagnose *B. canis* infections, additionally cross-reacts with IgM against *Escherichia coli* O116 and O157, *Salmonella* Urbana*, Francisella tularensis, Yersinia enterocolitica* O:9, *Afipia clevelandensis* and some other bacteria are witnessed. Lack of seroconversion could be the result of testing during the early phase of infection, due to blocking antibodies or prozone phenomenon. Such limitations can be avoided by modifications like addition of anti-human globulin, EDTA or 2-mercaptoethanol (Young 1991). SAT is less sensitive than microagglutination test (MAT) (Park et al. 2012). The comparison of SAT with 2-ME test exhibited lesser titer in 59.8% of human patients. However, equivalent results were observed in 2-ME test and EIA-IgG (Pabuccuoglu et al. 2011).

### 11.7. Brucellin test

The test is the old conventional way for testing of brucellosis in animals. This test is especially useful as a confirmatory test in unvaccinated animals and was an alternative test as per OIE (OIE 2009). It measures delayed type hypersensitivity reaction evident from increased thickness of skin. This test is more specific than common serological assays (Pouillot et al. 1997). However, its sensitivity is low which makes it a good test for herd but not for individual certification. However, since it takes a long time and effort, other rapid tests are preffered.

### 11.8. Enzyme-linked immunosorbent assay (ELISA) and its various versions

*Brucella* antigen detection by ELISA is a suitable alternative to culturing techniques, having 100% sensitivity and 99.2% specificity as per a study (Al-Shamahy and Wright 1998). Co-agglutination as well as antigen detection methods are considered appropriate techniques for *Brucella* specific antigen detection (Godfroid et al. 2010). The ELISA (IgG + IgM) and Brucella capt (immunocapture-agglutination) tests are reported as highly specific for human brucellosis diagnosis (Peeridogaheh et al. 2013). Combination of ELISA IgG and Brucellacapt can be an alternative to SAT (Aranís et al. 2008). Sensitivity of ELISA in acute brucellosis patients was found not higher than conventional assays such as microtiter-adapted Coombs test, titrated RBPT, microagglutination and Brucella capt (Gómez et al. 2008). Diagnostic sensitivity of bacterial culture and various serological techniques in brucellosis infected herds was analyzed; comparative assessment was performed among RBPT, CFT and indirect ELISA over 487 unvaccinated serum samples obtained from Turkey bovine herds with a history of abortion in last three years. Results advised to use RBPT and indirect ELISA both for better confirmation of *Brucella* infection (Gurbilek et al. 2017). Several types of ELISAs are there, *viz*., competitive and sandwich ELISAs, which could be useful for follow-up of cases of brucellosis (Ariza et al. 1992). Wang et al. (2015) developed a highly advanced version of a monoclonal antibody-based cELISA against LPS for the diagnosis of bovine brucellosis, which revealed higher specificity than the commercially available cELISAs and RBPT (Ahmed et al. 2011; Kirit et al. 2017). The analysis of the anti-*Brucella* antibody titers of naturally infected and vaccinated cattle by indirect ELISA, SAT, indirect hemagglutination assay and microtiter plate agglutination test revealed that the naturally infected animals presented much higher titers of agglutinating antibodies in comparison to the healthy vaccinated cattle (Mohan et al. 2016).

Praud et al. (2016) evaluated three commercially available cELISA kits and fluorescence polarization assay (FPA) for bovine brucellosis diagnosis and compared these with RBPT, CFT, indirect ELISA and FPA. The most sensitive tests were found as FPA, competitive ELISA and RBPT. CFT, SAT and RBPT were found to be highly specific. However, these three cELISA kits could not be recommended as a single screening test because of low specificity.

Simborio et al. (2015), in Korea, determined the efficacy of combined recombinant *B. abortus* outer membrane proteins 10, 19, 28 and individual recombinant outer membrane proteins for the diagnosis of brucellosis in cattle by ELISA, utilizing both SAT- positive and negative serum samples. The combined rOMP antigens revealed sensitivity, specificity and accuracy as 92.7, 98.7 and 96.0%, respectively. These rOMP combinations were thought to be promising vaccine candidates for development of highly effective vaccines in future.

*B. abortus* bacterioferritin (rBfr)-based ELISA determined the potential use of rBfr for the serological diagnosis of brucellosis in bovines (Hop et al. 2016). The rBfr detected antibodies against *Brucella* in positive sera in a dependent manner of SAT values; however, no immunoreaction was evident with negative serum samples. The rBfr was found promising for serodiagnosis of bovine brucellosis as accuracy, specificity, as well as sensitivity of rBfr were found to be very high (Hop et al. 2016).

Indirect ELISA measures IgG, IgM and IgA levels in serum, which is useful in clinical diagnosis of brucellosis. Indirect ELISA has gained higher promise in terms of both sensitivity and specificity as compared to SAT (Gad El-Rab and Kambal 1998; Almuneef and Memish 2003). This assay is considered highly sensitive for CNS brucellosis diagnosis (Ceran et al. 2011). The indirect ELISA sensitivity was compared with other conventional tests such as RBPT and 2-ME. The sensitivity as well as specificity of indirect ELISA was found to be 100% (Mirjalili and Lotfpouri 2016). The indirect ELISA showed an overall seroprevalence of 15.1% of brucellosis in buffalo in Punjab state of India (Islam et al. 2018). RBPT showed seropositivity of 41.3% for cattle serum samples, while indirect ELISA showed 54.4 and 45.7% samples as positive and negative, respectively (Sharma, Kalyani et al. 2015). In Bangladesh, researchers compared three tests, *viz.*, an IgG indirect ELISA, RBPT and SAT for the diagnosis of bovine brucellosis. A total of 1360 cattle serum samples were used and results depicted sensitivity of 84.6% while specificity was found 93.7%. They suggested using SAT and indirect ELISA together before making importation or culling of *Brucella*-positive animals and also suggested that brucellosis positive cattle should be eliminated out form the population as they are great risk for public health (Rahman et al. 2019).

### 11.9. Newer tools and modifications

Several field level tests, *viz*., lateral flow assay (LFA) and latex agglutination developed recently have been found to be easy to use and quick. It has been found that sensitivity as well as specificity of the LFA for culturally positive cases is more than 95% (Mizanbayeva et al. 2009; Marei et al. 2011). Similarly, sensitivity of 89.1% and the specificity as 98.2% were also reported (Abdoel and Smits 2007; Mantur, Bidari et al. 2007; Mantur, Amarnath et al. 2007). Both of these tests are suitable for field conditions as well as for hospitals in distant areas for use of healthcare workers (Abdoel and Smits 2007).

Smooth strains of *Brucella* result in production of very high level of antibody titers against the O-polysaccharide (McGiven et al. 2015). Gwida et al. (2016) studied the epidemiologic pattern of brucellosis in a cattle herd where multiple abortions were reported after regular vaccination with *B. abortus* RB51 vaccine. Spread of *Brucella* field strains was seen in the herd as evident by serological testing. Four strains of *Brucella* were isolated from aborted fetuses including one RB51 vaccine strain and three *B. abortus* field strains. The serologically positive cattle with positive RT-PCR results could possibly indicate *Brucella* field strain infection, while on the other hand the positive RT-PCR results from serologically negative cattle could be due to RB51 vaccine DNA in vaccinated cattle or due to the circulating field strain in cattle before the seroconversions (Gwida et al. 2016).

Pathak et al. (2018) evaluated the potential of Type IV Secretion System (T4SS), which is a major virulence factor of *Brucella,* as a serodiagnostic marker of *Brucella* infection. The immunological reaction of virB10 gene of *Brucella* T4SS recombinant antigen was evaluated with antisera following experimental infection of *B. melitensis* 16 M, BR31 and *Y. enterocolitica* O:9 in BALB/c mice. The recombinant antigen was also used to test 46 bovine serum samples. Significant antibody response against virB10 was evident in both experimental as well as natural hosts, which makes it a suitable target for serological diagnosis of *Brucella* infection.

Researchers are expressing interest to look into the possibilities of using circulating microRNAs (miRNAs) as clinical biomarkers (Ghai and Wang 2016). MicroRNAs are a group of small, non-coding RNAs which can significantly control genetic expression of immune components post-transcriptionally during infection to modulate the immune cell functions, either by activation or suppression of immune responses (Lawless et al. 2014). Infection with *B. melitensis* can modulate the *in vitro* expression of miRNAs impacting the immunological responses in host body (Rong et al. 2017). Circulating miRNA can be used as potential biomarkers for the non-invasive diagnosis of *B. abortus* infection in vaginal fluid and serum samples in water buffaloes (*Bubalus bubalis*). Findings of the study demonstrated alteration of 20 miRNAs, among which, 12 were upregulated and 8 were downregulated. In this way, study proved the diagnostic value of miRNAs for appropriate detection of *B. abortus* infection in water buffaloes (Lecchi et al. 2019).

Loop-mediated isothermal amplification (LAMP) of DNA as well as real-time PCR have been proved as significant, sensitive, quick and specific diagnostics for *B. abortus* and other *Brucella* spp. directly from clinical specimens (Karthik, Rathore, Thomas, Arun, Viswas, Agarwal et al. 2014; Karthik, Rathore, Thomas, Arun, Viswas, Dhama et al. 2014; Karthik et al. 2016; Patra et al. 2019). Real-time recombinase polymerase amplification (RPA) was developed targeting the *bcsp*31 gene and the sensitivity was found to be 94% (Qin et al. 2019). Real-time RPA targeting bp26 gene and lateral flow dipstick combined with RPA targeting *IS*711 of *Brucella* were developed and both assays were found to be specific in detection of *Brucella* spp. (Gumaa et al. 2019). Recently, polymerase spiral reaction for detection of *Brucella* spp. was developed which was 100 fold more sensitive than conventional PCR (Das et al. 2018). Rapid vertical flow technology using lipopolysaccharide of *Brucella* spp. was used for detection of anti-*Brucella* antibodies. The developed assay had an accuracy of 98% and hence can be used for early diagnosis of brucellosis at field level (Shi et al. 2020). Next generation sequencing of cerebrospinal fluid could be used for quick diagnosis of human neurobrucellosis enabling early treatment and better prognosis has been reported (Fan et al. 2018).

## 12. Treatment

Antibiotic treatment of brucellosis in domestic animals is often unsuccessful owing to intracellular survival of *Brucella* and its adaptability in the macrophages (Farid et al. 1961; Seleem et al. 2008). Low success rate of treatment and relapse of infection is very common in man. For brucellosis treatment in man, to prevent the side effects and emergence of resistance, combination of drugs should be selected wisely (Villate and Casallas 2020). Researchers used either ciprofloxacin and/or ceftriaxone as single drug for treatment of brucellosis cases but results were not promising (Doğanay and Aygen 1992; Lang et al. 1992). Combination therapies are preferred over monotherapy as it suggested the reduced chances of disease relapses (Feiz et al. 1973; Ranjbar et al. 2020). As monotherapy is not sufficient, hence for the treatment of uncomplicated brucellosis (without symptoms of endocarditis, spondylitis or neurobrucellosis) multi-drug therapy is preferred (Tuon, Cerchiari et al. 2017). Another regimen is use of doxycycline in dose of 100 mg twice daily orally along with 600–900 mg (15 mg/kg BW) of rifampin once a day for 6 weeks by oral route, amikacin two times a day for a week can also be included in the regimen to formulate triple drug therapy (Villate and Casallas 2020).

In an *in vitro* experiment performed to assess the sensitivity and efficacy of pefloxacin, lomefloxacin, meropenem and azithromycin against experimentally induced brucellosis, results demonstrated azithromycin was the most active drug followed by meropenem (Maletskaia 2002).

Dose regimen including doxycycline for six weeks in combination with rifampicin for six weeks duration or along with streptomycin for two to three weeks is also recommended (Colmenero et al. 1994; Ariza et al. 2007). A regimen comprising of doxycycline and streptomycin is considered to be the best therapeutic solution among others (Seleem et al. 2009). Individually streptomycin or doxycycline are not able to prevent the intracellular multiplication and adaptability of *Brucella* (Shasha et al. 1994). Though doxycycline-streptomycin regimen is thought to be the best; however, this has practical limitations, because the streptomycin has to be administered parentally for a period of three weeks. Another regimen, doxycycline for six weeks along with parental administration of gentamicin for a week is also considered suitable (Glynn and Lynn 2008).

When doxycycline and rifampin were compared for their efficacy in combination with co-trimoxazole in treating brucellosis patients, results revealed that the rate of disease relapse varies. Frequency of relapse was 1.96 times more when co-trimoxazole plus rifampin drug combination was used as compared to co-trimoxazole along with doxycycline (Roushan et al. 2004). Moreover, tauroursodeoxycholic acid or ginseng saponin fraction A has also been reported to inhibit intracellular replication of *Brucella* (Głowacka et al. 2018). Some researchers have used fluoroquinolones experimentally for the treatment of brucellosis and results do not advocate their use as first line of treatment (Pappas, Christou et al. 2006). Due to the peculiar nature of brucellosis, clinicians should have close collaboration with the microbiologist to diagnose, monitor and successfully treat human brucellosis (Mantur, Akki et al. 2004; Mantur et al. 2006; Mantur, Amarnath et al. 2007).

Effective management of treatment of bovine brucellosis is very important in infected dairy cattle herd, which has been described aptly and comprehensively by Singh et al. (2014). Treatment for brucellosis is typically long at least up to one month and could have several associated side effects. A novel anti-virulence compound, which leaves essential cell functions intact has been under advanced application research . Such an anti-virulence approach does not target normal functions, thus reduce the chances of antibiotic resistance significantly.

Researchers have reported a novel and successful immunotherapy for treatment of bovine brucellosis in cows by using RB51 phage lysates (as RL) and S19 (as SL). The cocktail of these two phage lysates (RL and SL) were injected subcutaneously in 2 mL-dose and even after 3 month-period of immunization by phage cocktail, blood samples were found negative for presence of *Brucella*. Among these two phage lysates, RL projected stronger cell-mediated immune response while SL stimulated higher level of humoral immune response. Results of the study are promising to encourage the use of bacteriophage lysates in treatment of bovine brucellosis (Saxena and Raj 2018).

## 13. Prevention and control

There is an increase in trade of animal products globally, which is also responsible for spread of various pathogens. Various regulations and guidelines should be meticulously followed during local, regional, national and international trade and transport of livestock and animal products. The transport of animal products should be done as per general principles and procedures provided in the International Zoo-Sanitary Code of the OIE in addition to the guidelines and prevalent practices in a locality. Various testing procedures for animals along with quarantine measures specified in this code should also be essentially followed (OIE 2016). Most South East Asian countries generally adopt the policy of Test-and-slaughter to eradicate the animal brucellosis (Zamri-Saad and Kamarudin 2016). This program could effectively reduce the incidence and prevalence of brucellosis; however, the disease could not be eradicated due to multiple reasons including difficulty to locate the brucellosis infected animals and inability to contain or regulate the movement of animals, purchase of animals without testing for brucellosis, and lack of education and interest of farmers regarding brucellosis. Additionally, an efficient system for control of animal brucellosis is dependent on several measures including robust surveillance mechanism to identify infected animals, prevention of spread from infected animals and herds to non-infected herds, removal of reservoirs of *Brucella* infection, preventive measures to stop re-introduction of the disease in a herd (Gwida et al. 2010).

Besides animal rearing and management practices, occurrence of brucellosis in cattle is also influenced by considering important key points such as proper certification of newly purchased heifers or bulls, their vaccination policy by using appropriate bacterial strain and assured culling of *Brucella* carrier animals. Hence, these factors should be incorporated while planning the *Brucella* control policies and campaigns. For successful control and eradication program of brucellosis, vaccination of heifers at large scale and practice of artificial insemination is recommended (de Alencar Mota et al. 2016). Semen introduction in the farm from certified brucellosis-free herd should be encouraged as it acts as important risk factor (Cardenas et al. 2019).

The epidemiological factors including animal reservoirs of infection are not considered after diagnosis, the priority becomes treatment of patients often with antibiotics, causing a setback to prevention and control measures (Corbel 2006; Hull and Schumaker 2018).

The purported health benefits of raw milk products and their consumption by rural population in brucellosis endemic areas has been a major route for higher incidence of human brucellosis that urgently needs accurate preventive strategies involving regular molecular detection and monitoring of the disease in livestock, their probable pathogen sources and implementation of hygiene measures during the dairy products processing (Dadar, Shahali et al. 2019).

Singh et al. (2018) estimated the impact of three different vaccination strategies for bovine brucellosis in India. These included mass vaccinations annually only for the replacement calves, mass vaccination of adult as well as young animal population at the beginning and subsequently vaccination of the replacement calves once a year, mass vaccination of replacements for one decade and followed by a test and slaughter policy for a decade. It could be shown that after following this approach for two decades, the prevalence of *Brucella* infection could very well drop below 2% in cattle and below 3% in case of buffaloes.

Proper surveillance programs often indicate the scenario of disease transmission and the pattern of zoonotic transmission of disease. To assess the correlation between bovine and human brucellosis, national surveillance program was performed in Korea on various cattle farms including beef cattle farm and beef slaughter houses during period of January 2004 to December 2014. It was concluded that there is a need of nation and worldwide comprehensive surveillance program for planning, control and eradication policies to decrease the brucellosis transmission from animals to human beings (Ryu et al. 2019). Nepomuceno et al. (2018) developed an individual based-mathematical model to show bovine brucellosis dynamics in Brazil. The results conclude that for eradication of the disease, approaches like isolation of infected animals and reduction of the size of population are essential.

## 14. Vaccination

The best way of prevention, control and eradication of brucellosis is by vaccination of all susceptible animal hosts at risk and elimination of positive animals in endemic areas (Briones et al. 2001). According to Susceptible-Exposed-Infectious-Recovered-Susceptible (SEIRS) model on brucellosis, it was observed that it takes 3.5 years to eliminate the disease from mixed cattle and sheep species farm endemic with *B. melitensis* following vaccination of sheep and cattle. Limiting the vaccination to sheep resulted in an increment in its elimination time to 16.8 years. Therefore, vaccination of cattle in endemic areas is utmost essential (Beauvais et al. 2016). Vaccine against brucellosis in animals plays a crucial role in the management of the disease in animals as well as in humans. The most common *Brucella* spp., *viz*., strain 19, RB51 and Rev1 are widely used as vaccine strains to protect against *Brucella* infection and related abortions in livestock. However, their use in other susceptible animals needs further studies and requires the development of novel effective vaccines in near future (Masjedian Jezi et al. 2019). *B. abortus* strains 19 and RB51 are very efficient and common vaccines being used against bovine brucellosis. *B. abortus* S19 was a result of natural attenuation lacking 720-bp region in the erythritol catabolic genes (Sangari et al. 1994; reviewed in Gheibi et al. 2018). The strain RB51 vaccine does not interfere with serodiagnostic results unlike strain 19 vaccine (Moriyón et al. 2004).

The best vaccine for the prevention of brucellosis in goats and sheep presently is *B. melitensis* strain Rev1 (Benkirane et al. 2014). The Rev1 vaccine is not recommended for administration during pregnancy as this vaccine exhibits high level of virulence resulting in induction of abortions. Moreover, the antibody response to vaccination interferes in diagnosis of natural infection. The *B. melitensis* Rev1 vaccine is used for small ruminants; however, this has yet to be completely evaluated for its use in bovines (Godfroid et al. 2010). However, complete and comprehensive evaluation of *B. melitensis* Rev1 vaccine has not been performed for use in cattle, but the Rev1 vaccine can be adapted for cattle vaccination in areas endemic for *B. melitensis* ( Corbel 1997; Banai 2002; Beauvais et al. 2016). Good animal management and surveillance are absolutely essential along with vaccination for potent and successful control of brucellosis (Morgan 1969). The economic analysis showed that a vaccination program covering the vaccination with S19 vaccine in 90% of the replacement heifers of 3–8 months of age provides excellent economic returns in a brucellosis vaccination program in bovines (Alves et al. 2015). *B. abortus* S19Δper vaccine, an intermediate rough strain, was found to be safe, immunogenic and also has the potential to be used as DIVA strategy vaccine for prevention and control of bovine brucellosis (Lalsiamthara et al. 2015).

Various other strategies have been evaluated for development of a safe and protective *B. abortus* vaccine. Expressed FliC protein of *Brucella* has been loaded on mannosylated chitosan nanoparticles and was used for immunizing mice. There was high level of IgG response, IFN-γ and IL-2 production. Similarly, immunized mice were found to be protective against *B. melitensis* biotype 1 strain 16 M and *B. abortus* 544 challenge (Sadeghi et al. 2020). Various recombinant proteins like Omp16, Adk, SecB, *etc*., were studied for their potential to be utilized as vaccine against brucellosis (Alizadeh et al. 2019; Huy et al. 2020). Subunit vaccines employing different proteins, like BP26, Omp25, L7/L12, *etc*., were found to be safer but the protection level was less. Recently a combined subunit vaccine of BP26, Omp25 and L7/L12 antigens was found to exhibit better protection against challenge than single antigen but lesser protection than *B. abortus* S19 (Gupta et al. 2019).

*B. abortus* vaccine should also be able to give cross-protection against *B. melitensis*. An influenza viral vector-*B. abortus* vaccine completely protected against abortions in pregnant heifers. An excellent level of cross-protection (90-100%) in the heifers, their calves or fetuses was observed upon challenge with *B. melitensis* 16 M. Influenza viral vector-*B. abortus* vaccine provided equivalent protection when compared with *B. abortus* S19 vaccine (Tabynov et al. 2015). These two vaccines were found to provide high degree of immunity against *B. melitensis* 16 M infection (Tabynov et al. 2015).

As preventive measures against brucellosis, besides conventional vaccines, new DNA vaccines and multivalent fusion DNA vaccine have also been developed (CDC 2017). They were found safe with good efficacy on laboratory and clinical trials. In this regard, immunization with multivalent DNA vaccines was done in BALB/c mice to assess the potential of immunogenicity. Multivalent DNA vaccines significantly induced high level of humoral immune response in terms of increased IgM, IgG, IgG2a, and enhanced cell-mediated immune response evidenced as high IFN-*γ* and lymphoproliferative response of splenocytes (Gomez et al. 2017). In another experiment, genes encoding for numerous open reading frames (ORFs) were selected from genomic island 3 (GI-3) of *B. abortus*. The antigens coding by these genes were responsible for bacterial virulence and intracellular survival of *B. abortus* in the host. When tested in mouse model, the DNA vaccine induced cytotoxic T cell and Th1 type immune responses, indicating the protective immune potential of such vaccine *via* immune cells (Gomez et al. 2018).

The approval of a vaccine and its associated adverse-reaction is not fixed at all time. In fact, it requires a series of critical judgments, which may include issues that may take over the course of many years (Grabenstein 2009). Brucellosis being a contagious and zoonotic potential disease has always led to the planning of improvised future vaccine, effective and regular vaccination programs and their safety evaluation from time to time. This has led to several improvements in the traditional vaccines and several different methods as well as routes of administration, which could establish better vaccine efficiency and reduce adverse effects. *Brucella abortus* strain 19 is the commonly recommended vaccine against animal brucellosis in India. However, several undesirable effects have seen to be associated with them when the vaccines were administered to animals or when humans were accidentally exposed. The occurrence of abortion-like adverse events was associated with three factors namely animal species, vaccination dose, and vaccination route, which was statistically authenticated (Xie et al. 2018). These results enable better understanding of brucellosis vaccine adverse effects and factors involved leading to enhancement of designing a more secure and effective vaccine. These advancements in brucellosis vaccine also requires knowledge of potential vaccine candidate, ability of DIVA, a vector vaccine for ease of administration as well several recombinant and subunit vaccines. Although, vaccination against brucellosis might be the most appropriate disease control measure but the persistence of pathogenic *Brucella* spp. in many other livestock or wildlife results in its failure or at best significantly decline in the human infections to some extent. Therefore, the development of novel vaccines that is highly effective and safe under field conditions, addresses the diversified host species along with improvement in other components of the regulatory program on brucellosis may significantly impact its prevalence worldwide (Olsen and Stoffregen 2005).

## 15. Conclusion and future perspective

Brucellosis is among the most prevalent animal and zoonotic diseases with worldwide occurrence. Brucellosis reveals mostly an endemic pattern of disease in developing regions especially sub-Saharan Africa. The prevalence of this disease is on the rise owing to numerous hygienic, social, economic, cultural and political factors. Brucellosis prophylaxis programs mainly depends on early, accurate and precise diagnosis of the disease. Brucellosis is diagnosed by history, symptoms of disease, bacteriological isolation and identification, serological tests, and various molecular tests including PCR-based assays. However, all the tests have some strengths and limitations. The major ailments caused by brucellosis include abortions, retained fetal membranes, endometritis, orchitis, epididymitis, *etc*., in animals and undulant fever in human beings. The disease causes colossal economic losses globally in terms of reduced animal health and production and effect on public health, yet robust surveillance, prevention and control measures are lacking.

Nation-wide comprehensive monitoring, surveillance programs with adequate funding in different geographical areas in all the countries where disease is prevalent should be conducted in order to assess the magnitude of the disease. Robust prevention, control and eradication strategies having collaboration of various departments should also be in force. Registration and proper identification of animals, excellent veterinary/medical services and adequate compensation are essentially required. The antibiotic treatment of brucellosis in domestic animals and humans is often unsuccessful or less successful. The ciprofloxacin and/or ceftriaxone as single drug for treatment of brucellosis cases were not reported promising. However, combination therapies are preferred over monotherapy for treatment of acute and chronic brucellosis, as reduced chances of disease relapses were reported. The multi-drug therapy including various antibiotics like doxycycline, streptomycin, gentamicin, rifampin and amikacin in different combinations are preferred to treat uncomplicated brucellosis cases and prevention of relapse.

Public awareness campaigns regarding disease symptoms in animals and man, epidemiology and source of infection, transmission of the disease and preventive measures in order to protect the public and for the planned control methodologies must be undertaken with full vigor. The major hurdle in controlling the disease in livestock includes meager budget allocation, lack of appropriate services to farmers having ailing animals along with restricted disease monitoring and surveillance to *B. abortus*. In case of humans, comprehensive educational and training programs are utmost necessary to control the disease in vulnerable groups like traditional small holders, healthcare providers and veterinarians. In this context, a financial investment-based collaborative approach of government, semi-government organizations, private industries and farmers is crucial in effective disease control strategies (Avila-Granados et al. 2019). Brucellosis is a major public health concern all around the world so a combined effort of veterinarians along with healthcare professionals is essential to curtail the disease.

Development of a vaccine which provides longer protection against all *Brucella* species is urgently required for prevention of the disease. Adequate funding and will are required at governmental level to combat this disease.
